# Supplementary material for: Patients’ Perspectives on Determinants Avoidable Hospitalizations: Development and Validation of a Questionnaire
Source: Int J Environ Res Public Health. 2022 Mar 7;19(5):3138. doi: 10.3390/ijerph19053138 (PMC8910657; doi:10.3390/ijerph19053138)
Supplement: Supplementary file 1 [file ijerph-19-03138-s001.zip › ijerph-1583861-supplementary.pdf]

Table S1. Final questionnaire about the determinants of potentially preventable hospitalizations

Good morning/good afternoon,

To start I would like to ask some questions about what happened for you for being admitted (to hospital) and I would like you to respond on a scale of *Not important* to *very important* you feel that ….

Individual	Not important	A Little Important	Not very nor Little Important	Important	Very Important	Do not Know/ Do not Respond
1.1. … not having support/help from friends or family was important for being hospitalized here?	□	□	□	□	□	□
1.2. … not having support/help from institutions with social services - residential help, meals at home, patients' associations etc. - was important for being hospitalized here?	□	□	□	□	□	□
1.3. … not having information about the symptoms of the disease was important for being hospitalized here?	□	□	□	□	□	□
1.4. … not having information about the prescribed medication was important for being hospitalized here?	□	□	□	□	□	□
1.5. … not having help to carry out the treatment successfully, not being able to get transportation or not having help to get medicine in case of emergency was important for being hospitalized here?	□	□	□	□	□	□
1.6. … not being able to evaluate the symptoms of the disease and to do something about them was important for being hospitalized here?	□	□	□	□	□	□
1.7. … not taking the medicines the way, they were prescribed was important for being hospitalized here?	□	□	□	□	□	□
1.8. … not fulfilling the recommendations regarding diet, exercise and consumption of alcohol, drugs and tobacco was important for being hospitalized here?	□	□	□	□	□	□
1.9. … having several chronic diseases was important for being hospitalized here?	□	□	□	□	□	□
1.10. … living in isolation was important for being hospitalized here?	□	□	□	□	□	□
1.11. … feeling anxiety and sadness was important for being hospitalized here?	□	□	□	□	□	□
1.12. … devaluing your illness was important for being hospitalized here?	□	□	□	□	□	□

Now, I am going to ask you some questions about health care and I ask you to answer me on a scale of *Never* to *Always*, with what frequency..:

Contextual	Never	Rarely	Sometimes	Frequently	Always	Do not Know/ Do not Respond
2.1. … do you feel that the health professionals responsible for your health care and treatments talk to each other about your needs?	□	□	□	□	□	□
2.2. … do the health services (medical services, nursing services, home care) are available when you need them?	□	□	□	□	□	□
2.3. … do the health services (medical, nursing, homecare services) meet your needs?	□	□	□	□	□	□
2.4. … do the health professionals responsible for your health care and treatments use words that you do not understand?	□	□	□	□	□	□
2.5. … do you trust the health professionals responsible for your health care and treatment?	□	□	□	□	□	□
2.6. … do the health professionals responsible for your health care and treatment give you enough time?	□	□	□	□	□	□
2.7. … is there a good relationship with the health professionals responsible for your health care and treatment?	□	□	□	□	□	□
2.8. … do you know which health service to use according to your health problem (hospital, family doctor or other health service)?	□	□	□	□	□	□
2.9. … do the health professionals responsible for your care and treatment talk to you about the different places you can go when you need care or treatment?	□	□	□	□	□	□
2.10. … do you feel that the health professionals help manage your illness?	□	□	□	□	□	□
2.11. … do you feel that the diagnosis of your disease was made at the appropriate time?	□	□	□	□	□	□
2.12. … when you contact the health professionals responsible for your health care and treatment (in person, by phone, by email), you get a response as fast as you need?	□	□	□	□	□	□
2.13. … when you contact the health professionals responsible for your health care and treatment, your values and traditions are considered?	□	□	□	□	□	□
